# Attitudes and practices regarding contraception among male students in a Nigerian tertiary educational institution: a cross-sectional study

**DOI:** 10.3389/frph.2024.1439900

**Published:** 2024-12-19

**Authors:** Awawu G. Nmadu, Jeremiah Musa, Istifanus A. Joshua, Adegboyega M. Oyefabi, Nafisat O. Usman, Bilkisu Nwankwo, Tukur Dahiru

**Affiliations:** ^1^ Department of Community Medicine, Faculty of Clinical Sciences, Kaduna State University, Kaduna, Nigeria; ^2^ Department of Community Medicine, College of Health Sciences, Ahmadu Bello University, Zaria, Nigeria

**Keywords:** contraception, male students, attitudes, practices, Nigeria, tertiary institution

## Abstract

**Objectives:**

Limited data exists on attitudes and practices of young men in tertiary institutions towards contraception. This study assesses attitudes and practices regarding contraception among male students in a tertiary educational institution in northwestern Nigeria, identifying factors influencing these attitudes.

**Methods:**

This cross-sectional study conducted from July to August 2021 at Kaduna Polytechnic, Nigeria, involved 160 male students chosen via multistage sampling. Structured questionnaires gathered information on socio-demographic characteristics, awareness, attitudes, and contraceptive practices. Analysis utilized SPSS version 23.0, employing descriptive statistics, bivariate, and multivariable logistic regression analyses to determine significant factors influencing contraception attitudes.

**Results:**

Findings revealed a mean respondent age of 25.4 ± 3.5 years, with most being single (71.3%) and 51.2% sexually active. While awareness of contraceptives was high (85.6%), almost half (46.7%) exhibited negative attitudes towards contraception. Common concerns included reliability, impact on sexual pleasure, and traditional gender norms. Only 35.8% had ever used contraception, primarily using withdrawal and male condoms. Significant factors influencing positive attitudes included being aged 18–24 years compared to 26–35 years (AOR = 2.66, 95% CI: 1.22–5.82).

**Conclusion:**

Culturally sensitive interventions are vital for improving negative attitudes and low contraceptive use among male Nigerian youth.

## Introduction

Sexually Transmitted Infections (STIs), Human Immunodeficiency Virus (HIV), prominent contributors to mortality and morbidity among young people, continue to escalate due to a high prevalence of risky sexual behaviour (1). Inconsistent condom use persists globally, alongside a rise in multiple sexual partnerships due to socio-cultural shifts and delayed marriages driven by higher education (2). In the context of sub-Saharan Africa, these health concerns are compounded by socio-cultural and religious factors that influence attitudes toward sexual health, particularly in conservative and patriarchal societies (3).

In many parts of sub-Saharan Africa, cultural and religious beliefs, particularly in predominantly Christian and Muslim communities, often discourage open discussions about sexual health and contraception. In Muslim regions of northern Nigeria, for example, strict interpretations of religious teachings can limit the acceptance of contraceptive use, especially for unmarried individuals, while gender norms often place the responsibility for reproductive health on women (4). Also, communities in the region often face their own cultural barriers to contraceptive use, often influenced by the community and local traditions, which prioritize abstinence or limit access to comprehensive sexual education (5).

These religious and cultural influences often result in significant gaps in sexual and reproductive health knowledge among young people, leading to risky behaviors, including early sexual debut, unprotected sex, and a reluctance to seek out information on contraception or STI prevention. This is reflected in data from across the region, where studies show that despite widespread awareness of HIV and other STIs, contraceptive use remains low, particularly among young adults, due to cultural and religious restrictions on contraception (1, 2). These factors contribute to the rapid escalation of STIs and HIV/AIDS among young people, particularly in tertiary institutions in Nigeria, where social freedom often leads to heightened sexual activity (6).

An analysis of data from the Nigeria Demographic and Health Surveys undertaken in 2008, 2013, and 2018 indicated that a significant number of unmarried youths (aged 15–24 years) participated in at least one risky sexual activity annually (7). Despite this, contraceptive usage remains low, with only 11.1% reported usage in 2018 (8). Alarmingly, about one-third of new HIV infections occur among adolescents and young people (9), highlighting the urgency of addressing contraceptive practices among this demographic. Despite the presence of studies on contraception among students in Nigeria, there is a glaring dearth of research exploring contraceptive attitudes and practices, specifically among male adolescents in tertiary institutions (10). During adolescence and young adulthood, valuable opportunities arise for introducing or improving information about reproductive health, developing healthy interpersonal skills, and influencing behaviours. Hence, intervention efforts specifically designed for males must be based on evidence that directly addresses their experiences and attitudes.

The limited data on men's views, attitudes, and behaviours concerning family planning (FP) in Nigeria underscore the urgency of addressing this gap, particularly among young men. Despite the recognized importance of men's involvement in reproductive health since the International Conference on Population and Development (ICPD), research and programs have historically focused on women's behaviours, neglecting the critical role of men in family planning decisions (11). Studies reveal significant gaps in young men's knowledge of contraceptive methods (12, 13), including emergency contraception, reflecting a broader need for comprehensive reproductive health education.

Students attending Nigerian tertiary institutions are identified as a high-risk group for reproductive health concerns, including engaging in unprotected sexual intercourse and having multiple sexual partners (14). These behaviors contribute to the rising prevalence of sexually transmitted infections (STIs), including HIV, and abortion-related complications, which pose significant health and social risks for young individuals. Male students, in particular, can play a critical role in contraceptive decision-making, yet their attitudes and behaviors regarding contraception remain underexplored.

The research question aims to uncover the attitudes of male students toward contraception, explore their contraceptive practices, delve into the reasons influencing these practices, and examine the factors influencing their attitudes. By addressing these questions, the research aims to shed light on misconceptions, sociocultural influences, and barriers to contraceptive use. The research intends to offer valuable insights for shaping health policies and practices. Ultimately, the goal is to encourage greater male involvement in contraceptive decision-making and the prevention of sexually transmitted infections, particularly during the pivotal college years.

## Methods

This cross-sectional study was carried out among male students aged 18–37 years from July to August 2021 at the Kaduna Polytechnic, North-western Nigeria. Kaduna Polytechnic, one of Nigeria's largest and most diverse tertiary institutions, enrolls over 30,000 students from across the country, representing a rich spectrum of ethnic, cultural, and socioeconomic backgrounds across its four colleges with more than 138 programs (15). Its standardized national admission criteria ensure comparability with other tertiary institutions. Its diversity and scale provide a robust context for examining youth attitudes and behaviors within Nigeria's tertiary education system. The study population consisted of undergraduate students present on campus during data collection. The study included all first to fourth-year, full-time, registered, male students of all age groups who were willing to participate in the study. Exclusion criteria included an unwillingness to participate and being a part-time student. The sample size was calculated to account for a 10% non-response rate (16), based on a standard normal deviate of 1.96, a contraceptive awareness of 0.92 (17), and a 5% margin of error, and was approximated to 160 using formula for cross -sectional study (*n* = z^2^pq/d^2^).

A multistage sampling approach was used to select participants. First, one college was randomly selected from Kaduna Polytechnic's four colleges. From the chosen college, two departments were randomly selected out of eight using simple random sampling (balloting). Class registers from departmental registration officers served as the sampling frame, detailing the number of male students in each level of which were: ND1 (84 and 152), ND2 (102 and 204), HND1 (152 and 202), and HND2 (114 and 213) across the two selected departments. Systematic sampling was used to select 20 students per level, with a sampling interval (k) calculated by dividing the total number of students in each level by 20. A random starting point was chosen, and every k-th student was selected. This approach resulted in a total sample of 160 students, ensuring equal representation across levels and departments.

A pretest was conducted with a sample of 16 students from a similar tertiary institution from a different city, Zaria in Kaduna State. The pretest aimed to assess the clarity, reliability, and validity of the study instruments. Based on feedback from this pre-test, minor adjustments were made to improve the wording of certain questions to ensure that they were easily understood by participants. The final version of the questionnaire was reviewed for content validity by subject matter experts in sexual and reproductive health, who provided feedback to ensure that it comprehensively addressed the key constructs being studied. Data collection utilized a semi-structured self-administered questionnaire adapted from a previous study (18) and comprising four sections: socio-demographic characteristics, awareness about contraception and sources of information, attitudes towards contraception, and contraceptive practices.

Attitudes towards contraception were assessed using a 5-point Likert scale, with scores ranging from 7 to 35 and median score of 23.0. Positive attitudes were defined as scores ≥ median, while negative attitudes were scores < median. Data analysis was done using IBM SPSS version 23. Descriptive analysis encompassed frequencies, percentages, mean and standard deviation. Binary logistic regression analysis determined the preliminary relationship between attitude toward contraception and independent variables such as age, marital status, year of study, and employment status. Additionally, significant factors (*p* < 0.25) from the binary logistic regression analysis were used in multivariate logistic regression (19). Crude and adjusted odds ratios were calculated and reported at 95% CI with a significance level of 0.05.

## Results

### Sociodemographic characteristics

A total of 160 students were recruited in the study with a response rate of 100%. The mean age of respondents was 25.4 ± 3.5 years. A majority of the respondents were in the Higher National Diploma 2 (HND2) level (58.8%), followed by the National Diploma 2 (ND2) level (21.3%). Only 28.8% of the respondents were married. Christianity (52.5%) and Islam (46.9%) were the predominant religions. About half of the respondents reported being sexually active (51.9%). A small proportion (5.6%) reported having children, and most respondents lived off-campus (66.3%) rather than in hostels (33.8%) (Table 1).

**Table 1:** Sociodemographic characteristics of male students in a tertiary educational institution, northwestern Nigeria 2021 (*N* = 160).

Variable	Frequency (*n*)	Percentage (%)
Age (in years)		
18–25	81	50.6
26–35	77	48.1
>35	2	1.3
Level of education		
ND1	21	13.1
ND2	34	21.3
HND1	11	6.9
HND2	94	58.8
Marital status		
Married	46	28.8
Single	114	71.3
Religion		
Islam	75	46.9
Christianity	84	52.5
Traditional	1	0.6
Sexually active		
Yes	83	51.9
No	77	48.1
Have children		
Yes	9	5.6
No	151	94.4
Place of residence		
Hostel	54	33.8
Off campus	106	66.3

### Awareness and attitudes towards contraceptives

Majority (85.6%) of the respondents had heard about contraceptives. Respondents primarily relied on friends (45.3%), the internet (44.5%) and health workers (44.3%) for contraceptive information, with mass media (32.1%) and relatives (28.5%) also being notable sources (Table 2).

**Table 2:** Awareness and sources of information about contraceptives among male students in a tertiary educational institution, northwestern Nigeria 2021.

Variables	Frequency (*n*)	Percentage (%)
Heard of contraceptives (*N* = 160)		
Yes	137	85.6
No	23	14.4
^a^Source of information on contraceptives (*n* = 137)		
Mass media	44	32.1
Relatives	39	28.5
Friends	62	45.3
Partners	33	24.1
Internet	61	44.5
Health workers	51	44.3
Others	8	5.1

^a^ Multiple response.

Overall, just over half (53.3%) of respondents exhibited positive attitudes towards contraception, while a considerable portion (46.7%) had negative attitudes (Table 3). Concerns about contraceptive reliability were prevalent, with 43.8% disagreeing and 19.0% strongly disagreeing about their reliability. Additionally, doubts about effectiveness are notable, with 45.9% believing that contraceptives have no significant effect or benefit. Concerns about their impact on sexual pleasure are expressed by 60.3% of respondents. Furthermore, 46.4% agree or strongly agree that contraceptives increase promiscuity. Health risks were also a concern, with 45.5% believing that contraceptives are harmful. Traditional gender roles persisted, as 60.2% considered contraception solely a woman's responsibility. Interestingly, while 75.9% agreed that couples should use contraceptives to prevent pregnancy if they don't desire it, 29.9% disagreed or were undecided (Table 4).

**Table 3:** Grading of attitude towards contraception among male students in a tertiary educational institution, northwestern Nigeria 2021 (*N* = 137).

Variable	Frequency (%)	Percentage (*n*)
Positive attitude	73	53.3
Negative attitude	64	46.7
Total	137	100

**Table 4:** Attitude towards contraception among male students in a tertiary educational institution, northwestern Nigeria 2021 (*N* = 137).

Variables	Strongly agree Freq(%)	Agree Freq(%)	Undecided Freq(%)	Disagree Freq(%)	Strongly disagree Freq(%)
Contraceptives are reliable	9 (6.6)	16 (11.7)	26 (19.0)	60 (43.8)	26 (19.0)
Contraceptives have no significant effect/benefit	15 (10.9)	48 (35.0)	43 (31.4)	25 (18.2)	6 (4.4)
Contraceptives decrease sexual pleasure	15 (10.9)	28 (20.4)	43 (31.4)	39 (28.5)	12 (8.8)
Contraceptives increase promiscuity	6 (4.4)	23 (16.8)	47 (34.3)	44 (32.1)	17 (12.4)
Contraceptives are harmful to health	11 (8.6)	30 (21.9)	44 (32.1)	32 (23.4)	20 (14.6)
Contraceptive is a woman's business; a man should not have to worry about it	33 (24.1)	49 (35.8)	22 (16.1)	18 (13.1)	15 (10.9)
Couples should use a contraceptive method to avoid getting pregnant if they do not want to.	5 (3.6)	11 (8.0)	17 (12.4)	41 (29.9)	63 (46.0)

### Contraceptive practices

Regarding respondents’ practice of use of contraception, 49 (35.8%) reported having used contraceptives before, while 88 (64.2%) had not. Of those who had used contraceptives in the past (*n* = 49), the withdrawal method was the most commonly reported, with 33 respondents (67.3%) indicating its use. Additionally, 20 respondents (40.8%) reported using male condoms, while only 2 (4.1%) reported vasectomy (Table 5).

**Table 5:** Practice of contraception among male students in a tertiary educational institution, northwestern Nigeria 2021.

Variables	Frequency (%)	Percentage (*n*)
Ever used contraceptives (*n* = 137)		
Yes	49	35.8
No	88	64.2
^a^Methods used in the past (*n* = 49)		
Withdrawal method	33	67.3
Male condom	20	40.8
Vasectomy	2	4.1

^a^ Multiple response.

**Figure 1:**
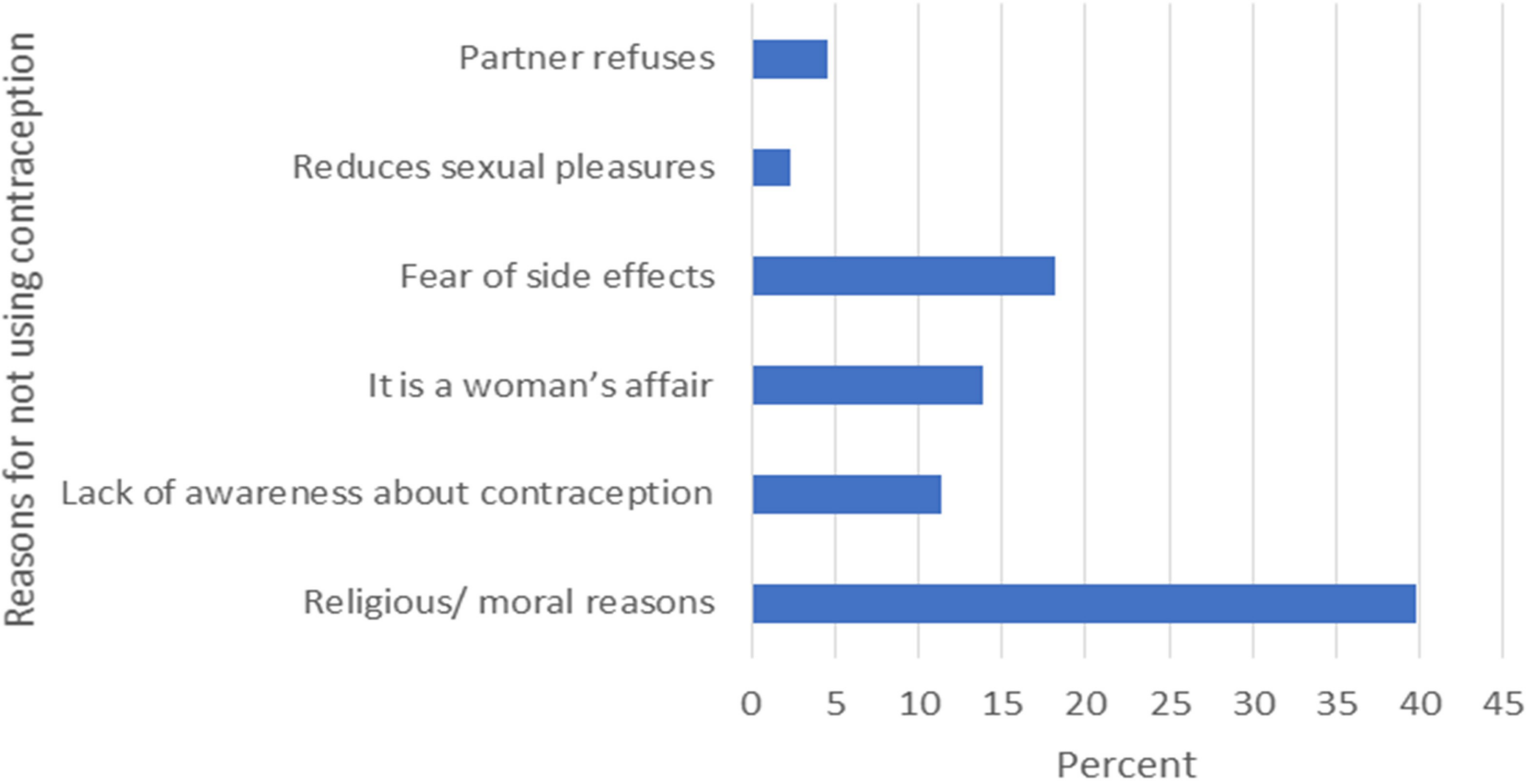
Reasons for not using contraception among male students in a tertiary educational institution, northwestern Nigeria 2021 (*n* = 88).

Figure 1 presents the reasons provided by respondents for not using contraceptives. The most frequently cited reason was religious or moral beliefs, with 35 respondents (39.8%) indicating this as their primary rationale. Lack of awareness about contraception was cited by 10 respondents (11.4%), while 12 respondents (13.8%) believed contraception was solely a woman's responsibility. Fear of side effects was another common concern, with 16 respondents (18.2%) mentioning it as a deterrent. A small percentage of respondents expressed concerns about the reduction of sexual pleasure (2.3%), and some reported that their partners refused to use contraceptives (4.5%).

### Factors associated with attitude towards contraceptives

Table 6 revealed that in the bivariate analysis, being in the younger age category (18–24 years) was associated with positive attitudes towards contraception, with an odds ratio of 0.28 (95% CI: 0.11, 0.70), compared to the older age category (25–34 years). Other variables such as ethnicity, level of study, marital status, religion, history of intercourse, having children, place of residence, history of contraceptive use, and current use of contraception did not show significant associations with attitudes towards contraception.

**Table 6:** Bivariate and multivariate logistic regression analysis of factors associated with attitude towards contraception among male students in a tertiary educational institution, northwestern Nigeria 2021 (*N* = 137).

Predictors	COR(95% CI)	AOR(95% CI)
Age (in years)		
18–24	0.28 (0.11, 0.70)	2.66 (1.22, 5.82)
25–34		
Sexual activity		
Sexually active	2.23 (0.86, 5.74)	–
Not sexually active		
Use of contraception		
Ever used contraceptives	0.50 (0.18, 1.42)	–
Never used contraceptives		
Had intercourse before		
Yes	0.661 (0.86, 4.34)	–
No		
Currently using contraception		
Yes	0.698 (0.67, 6.06)	–
No		

Only significant variables are presented after adjustment. Non-significant variables included ethnicity, level of study, marital status, and place of residence.

In the multivariate analysis, individuals aged 18–24 years were significantly more likely to hold a positive attitude towards contraception, with an adjusted odds ratio (AOR) of 2.66 (95% CI: 1.22–5.82). However, no significant associations were found for other variables after adjustment. Specifically, sexual activity, use of contraception, and having had intercourse before did not show significant associations with attitudes towards contraception.

## Discussion

The study's findings shed light on male students’ attitudes and practices regarding contraception, revealing a complex interplay of sociodemographic factors and cultural influences. Despite high awareness, negative attitudes towards contraception persist, driven by concerns about reliability and societal stigma. Traditional gender norms also impact perceptions, with contraception often viewed as solely a woman's responsibility. At the same time, there is acknowledgement of its importance in preventing unwanted pregnancies. These findings reflect broader cultural and socio-economic influences in shaping contraceptive attitudes and practices, consistent with studies in similar contexts (20, 21). These insights underscore the need for targeted educational interventions to address misconceptions and promote positive attitudes towards contraception among male students, ultimately improving reproductive health outcomes.

In terms of demographic traits, this study mirrors findings from a study conducted in Ghana on students’ attitudes toward emergency contraceptives (EC) (22). Both studies emphasized the youthful composition of respondents, with a majority falling within the 18–25 age range. Moreover, most participants in both studies were unmarried, which may influence their perceived need for contraceptives or emergency contraception. These findings highlight the importance of targeting young adults aged 18–25 with tailored reproductive health interventions. Understanding their attitudes toward contraception, including emergency contraceptives, is crucial for effective programing. Promoting contraceptive use among unmarried individuals can prevent unintended pregnancies, improving reproductive health outcomes for this demographic group.

The high level of awareness of contraceptives among respondents in this study aligns with previous studies, indicating a widespread awareness of contraceptives among young populations (18, 23, 24). However, the reliance on friends and the internet as primary sources of information, followed closely by health workers, highlights a notable departure from conventional channels like mass media and relatives (24, 25). This shift may reflect evolving trends in information consumption among the youth, potentially influenced by factors such as accessibility, peer influence, and digital literacy. However, despite this awareness, negative attitudes towards contraception prevailed in both studies. It is worrying that despite a high level of awareness of contraceptives, the majority of the male students in these studies displayed a negative attitude towards contraception for similar reasons. Significant proportions of the participants, respectively, indicating that contraceptives were unreliable, caused cancer, decreased sexual pleasure and increased promiscuity, which may make them not use contraceptives.

The prevalence of negative attitudes towards contraception observed in this study also echoes findings from studies conducted in Ghana, West Africa where similar concerns about contraceptives increasing promiscuity or reducing sexual pleasure were prevalent (22). Cultural and religious beliefs served as barriers to contraceptive use in both studies. In contrast to these, a study conducted in Cameroon showed students generally held positive more positive attitudes towards contraception than this study, although concerns about reliability, effectiveness, and health risks were notable (26). Traditional gender roles were also evident across these contexts, with contraception often viewed as solely a woman's responsibility. In Nigeria, negative attitudes were more pronounced, with a significant portion expressing doubts and concerns about contraception's reliability, effectiveness, and impact on sexual pleasure. Religious and moral beliefs played a significant role in shaping attitudes in both Nigeria and Ghana underscoring the need for targeted interventions to address misconceptions and promote positive attitudes and accurate knowledge to enhance uptake of contraceptives.

In contrast to the findings of our study, which highlighted a prevalence of negative attitudes towards contraception among male students, studies conducted in the United States and Ghana reported positive attitudes towards contraception among male college students (23, 24). These disparities suggest potential variations in attitudes towards contraception across different contexts and populations. Factors such as cultural norms, religious beliefs, and access to comprehensive sexual education programs may contribute to these differences. Comparing these findings underscores the importance of considering sociocultural influences and tailored interventions to address contraceptive attitudes and practices among male college students in diverse settings.

Furthermore, socio-economic and cultural factors also likely contributed to observed contraceptive practices in our study, similar to findings from the study in Cameroon. For instance, the preference for withdrawal as the most commonly used method may reflect cultural attitudes toward male-controlled contraception, limited access to modern contraceptives, and economic constraints. Regional comparisons, such as the DHS and PMA2020 survey data from 38 African countries indicated a higher prevalence of male condom use (27), suggesting that resource availability, health system infrastructure and and promotion strategies significantly influence contraceptive choices. Addressing these systemic discrepancies remain critical in improving reproductive health outcomes.

The reasons for not using contraceptives among male students in this study align with broader patterns observed in the literature, including religious or moral beliefs, lack of awareness, concerns about side effects, and partner refusal (18, 28). These findings underscore the need for targeted interventions addressing these barriers to contraceptive uptake. Interventions such as peer education programs, culturally sensitive counselling services, and comprehensive sexual education initiatives have shown promise in improving contraceptive knowledge, attitudes, and practices among college students (29).

Concerning sociodemographic factors and contraceptive practices, while our study identified a significant association between age and positive attitudes towards contraception on multivariate analysis, contrasting findings have been reported in studies by Wang et al. in China and in Cameroon (26), where older age groups exhibited more favourable attitudes. In Cameroon, both marital status and age emerged as influencing factors in attitudes towards contraception, with married and older individuals exhibiting more positive attitudes. Conversely, in Nigeria, age was associated with positive attitudes, with younger respondents showing greater acceptance, highlighting the importance of targeting younger populations with reproductive health education and interventions. Additionally, unemployment was prevalent in both studies, indicating potential socioeconomic factors influencing contraceptive practices. In contrast to this study, the Ghanaian study recognised the role of education and information dissemination in shaping attitudes to emergency contraceptives (24). The suggestion to utilize social media for information dissemination aligns with the need for innovative approaches to reach young people effectively. Interestingly, our study found no significant differences between married and unmarried participants in contraceptive attitudes or practices, suggesting that marital status may not be a decisive factor. However research by Ghosh et al. (20) and Riese et al. (21) highlight the complex interplay of individual, community, and societal factors in shaping contraceptive behaviours, suggesting that these factors are more influential than marital status alone. Therefore, while cultural context plays a central role, it is important to consider the intersectionality of factors in understanding contraceptive behaviours.

The finding that 2 (4.1%) out of 49 respondents reported undergoing vasectomy is notable, particularly within the context of acceptability of vasectomy in Nigeria and comparable settings. In Nigeria, vasectomy remains relatively uncommon among men due to factors such as religious and cultural beliefs, coupled with misconceptions surrounding vasectomy's impact on masculinity and sexual performance (30). Moreover, limited access to comprehensive sexual education exacerbates the dissemination of misinformation about vasectomy and other contraceptive methods (30, 31). Additionally, vasectomy's minimal presence in the method mix and family planning programming of most low- and middle-income countries (LMICs), including Nigeria, is another contributing factor to its low acceptance and utilization (32). In contrast, in countries such as Australia, United States, and New Zealand, vasectomy is more widely used, though research has shown that use has markedly declined globally, even as overall contraceptive use has risen in most countries (32). This acceptance is facilitated by a combination of factors including healthcare infrastructure, awareness, cultural acceptance, financial accessibility, and supportive policies contributes to the relatively higher usage rates of vasectomy in countries like the United States, New Zealand, and Australia compared to other regions (32). Addressing misconceptions, improving access to comprehensive sexual education, and incorporating vasectomy into family planning programming are essential steps toward promoting its acceptance and utilization in regions where it is currently underutilized.

While our study contributes valuable insights into the attitudes towards contraception among male students, informing targeted interventions for improving reproductive health outcomes, certain limitations exist. The cross-sectional design restricts causal inference, and reliance on self-reported data could introduce social desirability bias, where respondents may provide socially acceptable answers rather than true attitudes or behaviours. The sample, drawn from a single institution, limits generalizability of the findings to male students in other settings or regions. The structured questionnaire may have constrained responses, lacking qualitative depth to understand contextual influences on attitudes and practices. Further research exploring external factors using mixed-method approaches could deepen our understanding of the socio-cultural and economic influences on male contraceptive attitudes and practices. A multistage sampling approach was used to ensure diverse representation, with all selected students present during data collection, minimizing non-response bias. While this reduced potential biases from absenteeism, the timing and context of data collection may introduce inherent biases. These considerations are important when interpreting the generalizability of the findings.

Further research is warranted to explore the multifaceted factors influencing contraceptive behaviours in diverse cultural and geographical contexts. By building on existing literature and employing rigorous methodologies, future studies can inform the development of effective interventions aimed at promoting reproductive health and empowering male students to make informed choices regarding contraception. Targeted efforts are needed to address misconceptions, promote accurate information about contraceptive methods, and challenge traditional gender norms surrounding contraception. Comprehensive sex education programs, peer education initiatives, and culturally sensitive counselling services have shown promise in improving contraceptive knowledge, attitudes, and practices among young populations. Culturally appropriate interventions such as peer led education programs, leveraging social media for accurate information dissemination, and partnering with community and religious leaders to address cultural barriers are recommended. Additionally, incorporating tailored sex education into school curricula and providing personalized counseling services can help counter misconceptions, foster male involvement, and encourage positive attitudes towards contraception within the cultural context.

## Data Availability

The raw data supporting the conclusions of this article will be made available by the authors, without undue reservation.
